# Modeling reservoir management for malaria control in Ethiopia

**DOI:** 10.1038/s41598-019-54536-w

**Published:** 2019-12-02

**Authors:** Solomon Kibret, Darren Ryder, G. Glenn Wilson, Lalit Kumar

**Affiliations:** 1 Ecosystem Management, University of New England, NSW 2351 Armidale, Australia; 2 Present Address: Program in Public Health, University of California, Irvine, CA 92697 USA; 3 Present Address: Department of Biology, University of Southern Denmark, Campusvej 55, 5230 Odense M, Denmark

**Keywords:** Hydrology, Malaria

## Abstract

This study investigated how changes in reservoir water level affect mosquito abundance and malaria transmission in Ethiopia. Digital elevation models of three Ethiopian dams at lowland, midland and highland elevations were used to quantify water surface area and wetted shoreline at different reservoir water levels (70, 75, 80, 85, 90, 95 and 100% full capacity) to estimate surface area of potential mosquito breeding habitat. Reservoir water level drawdown rates of 10, 15 and 20 mm.day^−1^ were applied as scenarios to model larval abundance, entomological inoculation rate (EIR) and malaria prevalence at each dam. Malaria treatment cost and economic cost in terms of lost working days were calculated for each water level scenario and dam. At the lowland dam, increased larval abundances were associated with increasing reservoir water level and wetted shoreline area. In contrast, both larval abundances and area of wetted shoreline declined with increasing reservoir water level at the midland and highland dams. Estimated EIR, malaria prevalence, malaria treatment cost and economic cost generally decreased when the water level drawdown rate increased from 10 to 15 and 20 mm.day^−1^ irrespective of reservoir water level. Given the expansion of dam construction in sub-Saharan Africa, incorporating malaria control measures such as manipulating drawdown rates into reservoir management has the potential to reduce the malaria burden and health care costs in communities near reservoirs.

## Introduction

Despite a significant malaria decline in recent years^1^, sub-Saharan Africa continues to represent a disproportionately high share (92%) of the global malaria burden (200 million annual cases)^2^. Malaria is not only a major public health challenge but also a key economic impediment for the region. The annual economic cost of malaria in Africa was estimated to be US$12 billion, including the costs of health care, working days lost due to sickness, days lost in education, decreased productivity due to hospitalization, and loss of investment and tourism^3^. Consequently, the annual economic growth of countries with malaria transmission has historically been lower than countries without malaria^4^. Malaria is a major public health challenge in rural farming communities and transmission generally coincides with the planting and harvesting seasons, and hence affects agricultural productivity^5^.

To improve the livelihoods of rural communities and to foster regional economic development, sub-Saharan Africa (SSA) has recently embarked on a new era of water resources development that involves extensive dam construction^6^. However, the impact of dams on increasing rates of malaria transmission has raised concerns regarding the sustainability of these infrastructures. A recent study revealed that over one million annual malaria cases are associated with dams in this region^7^. A number of studies across SSA showed that dams increase malaria incidence by creating breeding habitats for malaria vector mosquitoes adjacent to human settlements^8–14^. The two principal African malaria vector mosquitoes, *Anopheles gambiae* and *An. arabiensis*, thrive in shallow shoreline puddles around reservoirs^11,15^.

Construction of dams for irrigation and hydroelectric generation could thus pose substantial public health challenge unless appropriate measures are put in place. Over 2000 large dams currently exist across SSA, and an additional 200 dams are under construction^16^. To deal with malaria around these economically important infrastructures, Africa requires a set of complementary measures for malaria control interventions, tailor-made to address local circumstances. Vector control measures that involve the use of long-lasting insecticide treated bed nets (LLIN), indoor residual spraying (IRS) and larval source management (LSM) are the major malaria intervention tools used in endemic countries^17^. LLIN and IRS are the most common and widely practiced control measures that target mosquito vectors feeding or resting indoors. However, the challenges of insecticide resistance and high numbers of outdoor host-seeking^18,19^ and resting^20^ vector mosquitoes, coupled with high operational costs of LLIN and IRS^21^, have recently led to a renewed interest in LSM as a viable intervention.

LSM is the management of water bodies that serve as potential mosquito breeding habitats to prevent the development of immature stages. Control of immature mosquito populations is advantageous because the larvae are usually spatially concentrated, relatively immobile, and occupy confined habitats compared with adult stages that can rapidly disperse over large areas. Effective larval control minimizes the cost of adulticides, and is cost-effective and environmentally friendly^22,23^.

Around reservoirs, LSM through water level manipulation can render conditions unfavorable for mosquito larvae to complete their aquatic development^24,25^. In the Tennessee Valley of the United States, reservoir water management significantly reduced the development time of mosquito larvae around the reservoirs^26^. During the malaria mosquito production period, cyclical fluctuations (0.3 m of vertical change per week) of reservoir water levels were applied at intervals of seven to ten days to effectively reduce larval mosquito populations. However, such techniques have been poorly investigated for their application in Africa, despite the potential increase of malaria transmission with the projected levels of dam development.

With the current extensive dam construction in SSA, reservoirs could continue to increase malaria transmission. To mitigate this challenge, optimized reservoir management is crucial to supplement existing malaria control tools. The present study investigated how reservoir water level changes affect mosquito breeding and malaria transmission around three dams located in different eco-epidemiological settings in Ethiopia. First, we modeled the surface areas of wetted shoreline at different reservoir water levels that commonly occur during malaria transmission seasons. Estimates of reservoir-scale larval productivity were used to calculate the malaria transmission intensity (i.e. entomological inoculation rate), malaria treatment cost and economic cost related to lost working days for different water level drawdown rates and reservoir capacity scenarios.

## Methods

### Study area

**Figure 1:**
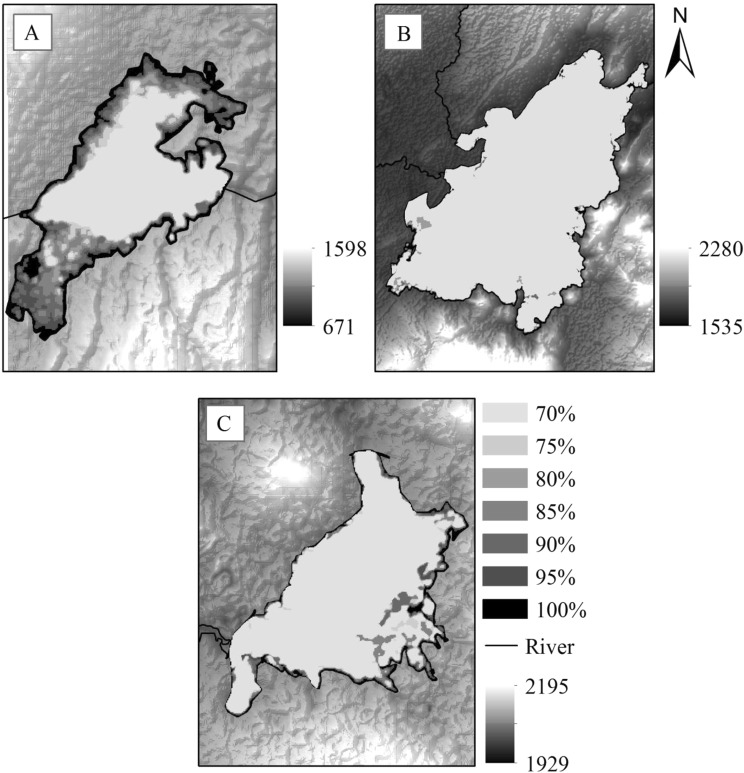
Reservoir models showing reservoir shoreline at different water level capacity. (**A**) Lowland dam, (**B**) midland dam, and (**C**) highland dam.

This study was conducted around three large dams in Ethiopia: the Kesem Dam (referred as the lowland dam), Koka Dam (referred as the midland dam) and Koga Dam (referred as the highland dam) (Fig. 1). A recent study classified ecological settings in Africa, using climate and elevation characteristics, as lowland (<1000 m above sea level, m asl), midland (1000–1700 m asl) and highland (>1700 m asl)^27^: the present study adopted the same definitions to classify the three study dams.

The Kesem Dam (975 m asl) has a crest height of 25 m, and a maximum storage capacity of 500 million m^3^. The surface area of the reservoir at full capacity is 200 km^2^. An estimated the length of the reservoir at full storage capacity is 55.4 km. An estimated 89,000 people live within 10 km of the Kesem reservoir^28^. The primary purpose of the dam is to irrigate 20,000 ha of sugarcane plantation downstream. The closest village to Kesem reservoir is located 450 m from the shoreline when the dam is full. The Koka Dam (1551 m asl) has a crest height of 42 m, and a full water storage capacity of 1188 million m^3^. The surface area of the reservoir at full capacity is 236 km^2^ and the length of the reservoir at full storage capacity is 86 km. The primary purpose of the Koka Dam is to generate 43.2 MW of electricity. The closest village to the Koka reservoir is only 300 m from the shoreline. An estimated 67,865 people live within 10 km of the Koka reservoir^28^. The Koga Dam (1980 m asl) has a storage capacity of 83.1 million m^3^ and surface area of 175 km^2^. The length of the reservoir shoreline at full capacity is 120 km. The closest village to the Koga reservoir is only 300 m from the shoreline. An estimated 45,366 people live within 10 km of the Koga reservoir^28^. The rainy season in the three dams extends between June and August. Reservoirs fill during this period and they often reach full capacity at the end of August. The dry season lies between January and March. Further characteristics of the study area are presented in Kibret *et al*.^29^.

### Data sources

#### Digital elevation data

A high resolution (30 m × 30 m) digital elevation model (DEM) was obtained for each of the three dam sites from the Ethiopian Ministry of Water Resources. Shapefiles for reservoir shorelines at full supply level were created by digitizing Google Maps and importing to ArcGIS. Using the crest elevation as a reference for full capacity, each dam was modeled to show scenarios for reservoir surface area and shoreline perimeter at different reservoir water capacities. Data from previous work^29^ demonstrated that 56–71% of annual malaria cases around the three dams occur between September and December (Table 1). This period is when reservoir management has the maximum potential to suppress larval development and malaria transmission. During this period, reservoir capacity averaged between 71–95% across the three dams (Table 2). Average reservoir water level during the main malaria season (in 2010–2014) ranged from 981.4–984.4 m asl at the lowland dam, 1559.5–1563.2 m asl at the midland dam, and 1991.2–1995.5 m asl at the highland dam (Table 2). Thus, reservoir surface area and shoreline perimeter were modeled at 70, 75, 80, 85, 90, 95 and 100% of full capacity to quantify reservoir-scale mosquito larval abundances.

**Table 1:** Annual number of malaria cases around the study dams, 2010–2014. [Data source: Kibret *et al*.^28^].

		Lowland dam	Midland dam	Highland dam
2010	Annual malaria cases	899	495	137
	No. cases in Sep-Dec	562	325	97
	% of annual	63%	66%	71%
2011	Annual malaria cases	1099	686	261
	No. cases in Sep-Dec	614	420	139
	% of annual	56%	61%	53%
2012	Annual malaria cases	1199	599	183
	No. cases in Sep-Dec	683	362	117
	% of annual	57%	60%	64%
2013	Annual malaria cases	1358	599	183
	No. cases in Sep-Dec	761	362	117
	% of annual	56%	60%	64%
2014	Annual malaria cases	1358	898	249
	No. cases in Sep-Dec	761	541	152
	% of annual	56%	60%	61%

**Table 2:** Mean elevation and reservoir water level during the main malaria transmission, 2010–2014. [Data source: Kibret *et al*.^29^].

Year	Elevation* (and % reservoir water level)		
	Lowland dam	Midland dam	Highland dam
2010	984.4 (76–93%)	1560.7 (73–95%)	1995.5 (82–94%)
2011	981.4 (72–91%)	1561.2 (73–91%)	1994.3 (78–92%)
2012	980.6 (71–89%)	1562.1 (75–92%)	1992.1 (74–92%)
2013	982.3 (72–92%)	1559.5 (72–90%)	1991.8 (74–93%)
2014	981.9 (73–94%)	1563.2 (76–93%)	1991.2 (72–91%)

^*^Elevation is in meters above sea level.

#### Water level drawdown rates

Four water level drawdown rates (0, 10, 15, 20 mm.day^−1^) based on previous experimental work^30^ were used to model the impact of different water drawdown rates on mosquito larval and adult abundance, and malaria risk. These operational regimes were confirmed to have significant impact on malaria mosquito abundance based on field study.

#### Mosquito data

Data from a previous study^29^ were used to estimate larval vector mosquito abundance (i.e. *An. arabiensis*) around the three study dams at each reservoir capacity scenario during the peak malaria season (September-December). Maximum adult mosquito vector travel distances of 5 km from shoreline puddles were used to estimate the risk of malaria transmission to villages within the dispersal range^31^.

#### Population data

Population data of villages within a 5 km radius of each reservoir at 70, 75, 80, 85, 90, 95 and 100% of full capacity were obtained from the Ethiopian Central Statistics Agency^28^. The villages were georeferenced and the population data imported to Microsoft Excel and ArcGIS.

#### Malaria treatment cost

To estimate malaria treatment costs associated with each water level scenario, we used published data from Deressa *et al*.^32^ that reported the economic costs for malaria treatment in rural Ethiopia. The same treatment costs were applied to the three study dams.

#### Economic cost

To estimate the employment person-days lost due to malaria, data from Deressa *et al*.^32^ were used, which found that the mean number of days lost per hospitalized patient in rural Ethiopia was 14.5 days per malaria patient and 17.1 per caretaker.

### Data analysis

#### Modeling reservoir parameters

The wetted shoreline perimeter (<0.5 m depth) for each water capacity scenario and reservoir was estimated as potential mosquito breeding habitat. Each polygon (30 m × 30 m) of the wetted shoreline was counted on ArcGIS to determine area and perimeter. Area of wetted shoreline for each reservoir capacity scenario that potentially supports larval breeding was calculated by multiplying the shoreline perimeter in each reservoir water level scenario by the estimated shoreline habitat that potentially supports mosquito breeding (i.e. shoreline puddles with <0.5 m depth)^29^.

#### Estimating malaria vector larval abundance associated with each water level scenario

Using data from previous work^29^, mosquito larval abundance was calculated for each reservoir at each water level scenario and reservoir capacity. The study found that anopheline larval density (no. larvae per m^2^) declined by 30%, 70% and 84% compared to the control (i.e. no change in water level) when water level drawdown rates of 10, 15 and 20 mm.day^−1^ were applied to *in situ* experimental dams. Each percentage reduction in larval density was applied to all three dam sites. For each dam, the reservoir-scale anopheline larval abundance (without optimizing the water levels) was estimated by multiplying the observed anopheline larval density^29^ by the area of potential mosquito breeding shoreline habitat in each reservoir capacity scenario (as estimated above). Anopheline larval abundance (LA) was also estimated for each of the three selected water level drawdown rates (10, 15 and 25 mm.day^−1^) for each dam as: LA = LD * R * A, where LD is the anopheline larval densities obtained from previous field survey^29^ around each of the three dams (i.e. 10.8 ± 3.7 (SE), 5.1 ± 1.1 and 0.5 ± 0.2 for the lowland, midland and highland dams, respectively; R is the factor by which the larval densities reduce when water level rates of 10, 15 and 20 mm.day^−1^ are applied (R is 0.30, 0.70 and 0.16, respectively); and A is area of potential mosquito breeding shoreline habitat in each reservoir water level scenario.

#### Estimating human population around reservoir shoreline at different water level scenarios

The total human population living within a 5 km radius of the reservoir shoreline was estimated for each water level scenario by measuring the distance of each village (i.e. the center of the village) from the shoreline in each scenario using ArcGIS.

#### Estimating malaria risk

Entomological Inoculation Rate (EIR) is a more direct measure of malaria transmission intensity than traditional measures of malaria prevalence or hospital-based measures of infection or disease incidence^33^. EIR from larval abundance was estimated using the equation derived by Gu and Novak^34^. The conventional formula for EIR^35^ is the product of human-biting rate (*ma*, where *m* is the number of host-seeking mosquitoes per person and *a* is the man-biting tendency of individual mosquito species) and the proportion of sporozoite infected mosquitoes (*s*) as EIR = *mas*. Gu and Novak^34^ rearranged this formula to determine EIR from larval mosquito abundance as:

$${\rm{EIR}}=\gamma P{e}^{-dT}as,$$

where γ is the base level of emerging female mosquitoes, *P* is larval productivity (no. larvae per m^2^), *d* is daily mortality rate of adult mosquitoes, *T* is the extrinsic incubation period, *a* is man-biting habit (same as the Human Blood Index), and *s* is the proportion of sporozoite infected mosquitoes. The values *γ*, *d* and *T* were taken from a previous study that estimated these values from robust data in Africa^35^. The values for *a* and *s* were adopted from previous studies in the same area^29^. The difference in EIR between water level drawdown rates was compared at each dam by Analysis of Covariance (ANCOVA) using SPSS version 22.

#### Estimating malaria cases

To estimate the number of malaria cases that arise from the EIR, a formula derived by Smith *et al*.^33^ was adopted. The malaria prevalence rate (PR) was computed as:

$$PR=1-{(1+\frac{{\rm{b}}\varepsilon }{rk})}^{-\kappa }$$

where ε refers to EIR, *b* is transmission efficiency (i.e. the probability that a bite by an infectious mosquito results in an infection), 1/*r* the expected time to clear each infection, and *k* is a constant that takes into account the heterogeneous infection (i.e. the fraction of all infections received by the subpopulation that is infected most). Smith *et al*.^33^ estimated that 1/*k* is 4.2 and *b/r* is 0.45 using an extensive African dataset, and these values were used in the present study. After estimating the malaria prevalence rate, the total number of malaria cases was calculated by multiplying PR by the total population around the reservoir shoreline (<5 km) for each water level and reservoir capacity scenario at each dam.

#### Malaria treatment cost

The malaria treatment cost was estimated by multiplying the US dollar cost of malaria treatment at health facilities based on data from Ethiopia^31^ by the number of malaria patients estimated in each reservoir water level drawdown and water level scenario. The dollar value between 2007 (the year where the previous study by Deressa *et al*.^32^ was conducted) and “2015 (present study) was adjusted based on the” World Bank’s Consumer Price Index^36^.

#### Economic cost

The total number of days lost per hospitalized malaria patients around each dam for each reservoir capacity scenario and modelled rates of water level drawdown was calculated by multiplying the number of malaria patients in each scenario by the number of days lost per hospitalized malaria patient (14.5 days; based on Derressa *et al*.^32^).

#### Model validation

Data of reservoir water-level data and mosquito larval abundance for all the three dams for the period between July 2013 and May 2014 was obtained from previous work^29^ for model validation. Actual and predicted larval abundances were plotted and compared.

## Results

### Reservoir models

As reservoir capacity increases from 70 to 100%, the perimeter of wetted shoreline and area of larval habitat increased at the lowland dam, but decreased at the midland and highland dams (Fig. 1; Table 3). At the lowland dam, the wetted shoreline area and perimeter was highest when the reservoir was 90% capacity. In contrast, the wetted shoreline area and perimeter decreased as reservoir capacity in the midland and highland dam approached full capacity. The midland dam generally had the highest perimeter and area of wetted shoreline, followed by the highland and lowland dams.

**Table 3:** Reservoir model parameters and malaria vector larval abundance.

	Reservoir water level	Shoreline Perimeter (m)	Larval habitat area (m^2^)	Total no. larvae at reservoir scale	Larval abundance - water level drawdown rate models		
					10 mm.day^−1^	15 mm.day^−1^	20 mm.day^−1^
Lowland dam	70%	35,083	17,542	190,107	133,075	57,032	8449
	75%	40,182	20,091	189,448	132,614	56,834	8420
	80%	43,581	21,791	216,983	151,888	65,095	9644
	85%	53,900	26,950	235,337	164,736	70,601	10,459
	90%	61,245	30,623	291,060	203,742	87,318	12,936
	95%	58,696	29,348	330,723	231,506	99,217	14,699
	100%	55,417	27,709	316,958	221,871	95,088	14,087
Midland dam	70%	1,110,842	555,421	3,379,082	2,365,357	1,013,724	540,653
	75%	1,110,842	555,421	2,832,647	1,982,853	849,794	453,224
	80%	860,383	430,192	2,832,647	1,982,853	849,794	453,224
	85%	860,383	430,192	2,193,977	1,535,784	658,193	351,036
	90%	648,901	324,451	2,193,977	1,535,784	658,193	351,036
	95%	648,901	324,451	1,654,698	1,158,288	496,409	264,752
	100%	487,926	243,963	1,654,698	1,158,288	496,409	264,752
Highland dam	70%	76,875	76,875	33,778	23,645	10,133	5,405
	75%	69,825	69,825	38,438	26,906	11,531	6,150
	80%	69,825	69,825	34,912	24,439	10,474	5,586
	85%	64,216	64,216	34,912	24,439	10,474	5,586
	90%	55,235	55,235	32,108	22,476	9,632	5,137
	95%	48,582	48,582	27,617	19,332	885	4,419
	100%	42,728	42,728	24,291	17,004	7,287	3,887

### Larval vector abundance

The total area of potential mosquito breeding habitat around the shoreline increased at the lowland dam but decreased at the midland and highland dams as the reservoir approached full capacity (Table 3). At the lowland dam, the reservoir-scale area of mosquito larval habitat increased as the reservoir capacity increased, with the largest area recorded at 90% capacity. Consequently, reservoir-scale larval mosquito abundance increased as the reservoir approached 90% capacity and declined slightly as the dam reached full capacity.

Faster rates of water level drawdown were associated with lower reservoir-scale total larval abundances at all dams (Table 3). At the lowland dam, water level drawdown rates of 10, 15 and 20 mm.day^−1^ were associated with a 24%, 54% and 72% reduction, respectively, in larval abundance compared with those in constant water level scenarios. At the midland dam, these water level drawdown rates were associated with a 48%, 78% and 88% reduction in larval abundance compared to a constant water level scenario. Similarly, a 17%, 65%, 81% reduction in larval abundance was found at the highland dam when the reservoir was simulated at water level drawdown rates of 10, 15, and 20 mm.day^−1^, respectively.

### Entomological inoculation rate (EIR)

**Figure 2:**
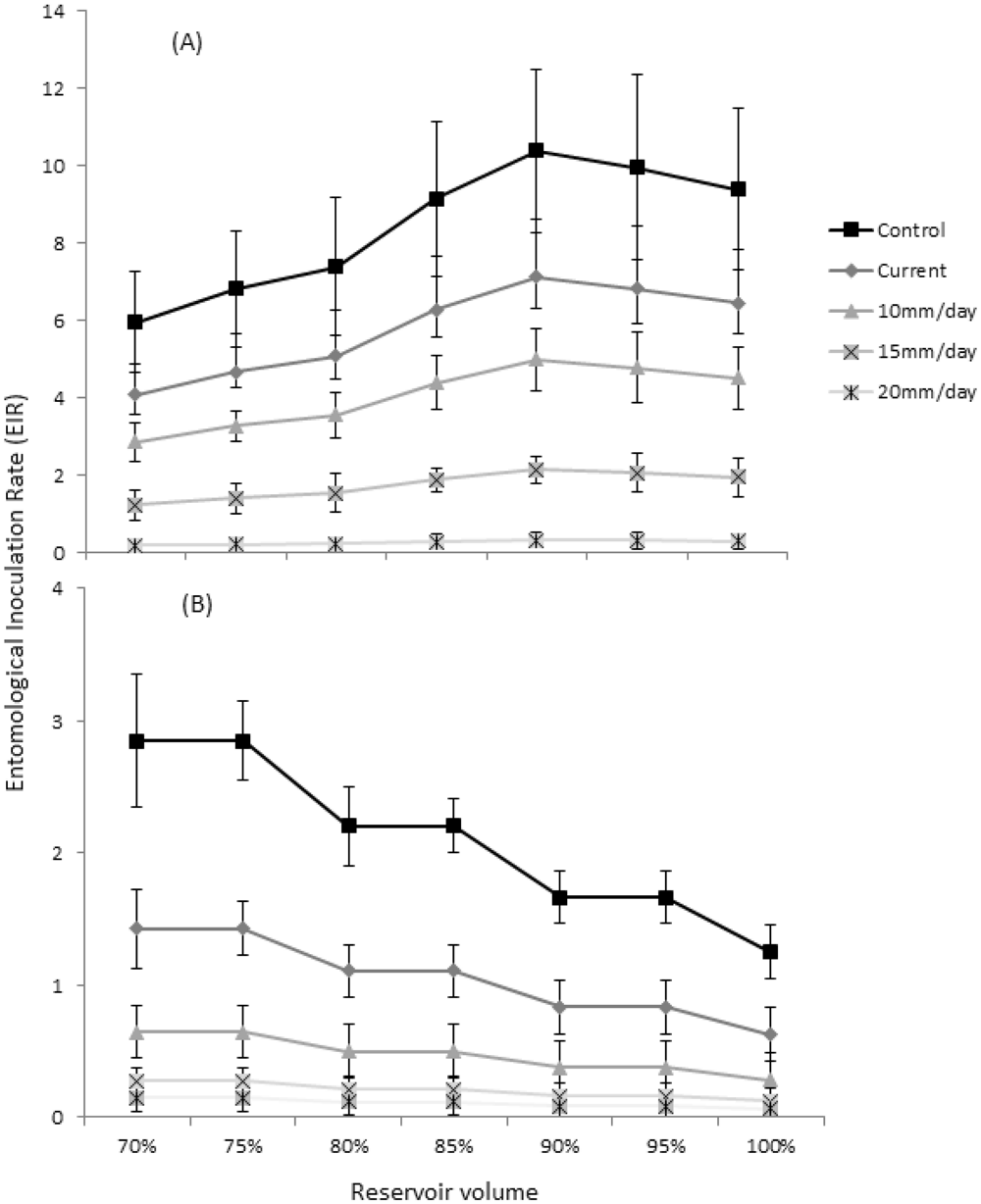
Estimates of Entomological Inoculation Rate (EIR) at the (**A**) lowland and (**B**) midland dams for different reservoir water level scenarios and water level drawdown rates.

The EIR mirrored the trend in larval and adult abundances in the lowland and midland dam (Fig. 2). EIR was not estimated for the highland dam as no sporozoite-infected mosquitoes have been reported in this eco-epidemiological region^29^. The EIR was significantly higher at the lowland dam than the midland dam (F = 6.73; df = 1; *P* < 0.01), ranging from an EIR of 4.2–7.1 at the lowland dam and 0.6–1.7 at the midland dam. Water level drawdown rates of 10, 15, and 20 mm.day^−1^ were associated with a 19%, 48% and 65% reduction in EIR at the lowland dam and a 40%, 71% and 82% decline at the midland dam, respectively.

### Malaria burden

The number of malaria cases dropped considerably at the lowland dam from its current 33,700 to 25,700, 15,600 and 9,400 as the water level drawdown rate increased from 10 to 15 and 20 mm.day^−1^, respectively (Table 4). Compared to the current malaria burden, these drawdown rates are associated with a 24%, 54% and 72% reduction in the number of malaria cases. At the midland dam, the number of malaria cases estimated at current, 10, 15 and 20 mm.day^−1^ water level draw down rate was 11,700, 6,100, 2,600 and 1,500, respectively, revealing a potential decrease of 47%, 78% and 87%, respectively.

**Table 4:** Estimated number of malaria cases at different water level drawdown rate scenarios.

	Reservoir water level	Malaria cases - Water level drawdown rates				% Change from current		
		Current	10 mm.day^−1^	15 mm.day^−1^	20 mm.day^−1^	10 mm.day^−1^	15 mm.day^−1^	20 mm.day^−1^
Lowland	70%	3,092	2,365	1,439	868	24	53	72
	75%	3,539	2,707	1,646	993	24	53	72
	80%	3,837	2,934	1,784	1,076	24	54	72
	85%	4,742	3,626	2,203	1,327	24	54	72
	90%	5,387	4,118	2,501	1,506	24	54	72
	95%	5,163	3,947	2,398	1,444	24	54	72
	100%	4,876	3,728	2,264	1,364	24	54	72
	Total	33,738	25,798	15,677	9,450	24	54	72
Midland	70%	1,841	965	411	234	48	78	87
	75%	1,841	965	411	234	48	78	87
	80%	1,428	750	321	184	47	78	87
	85%	1,428	750	321	184	47	78	87
	90%	1,080	569	245	141	47	77	87
	95%	1,080	569	245	141	47	77	87
	100%	815	431	187	109	47	77	87
	Total	11,707	6,148	2,629	1,503	47	78	87

### Malaria treatment cost

Similar to the number of malaria cases, the cost of malaria treatment was generally higher at lower rates of water level drawdown (Table 5). At the lowland dam, the cost of malaria treatment was USD 46,000, 41,000, 25,000 and 15,000 at current, 10 mm.day^−1^, 15 mm.day^−1^ and 20 mm.day^−1^ water level scenarios, respectively. Compared with current, costs declined by 11%, 46% and 67% when 10, 15 and 20 mm.day^−1^ water level rates were applied. At the midland dam, the total cost of malaria treatment in reservoir communities was estimated to be USD 15,000. The cost declined by 47% (USD 7,900), 77% (USD 3,400) and 87% (USD 1,963) at water level drawdown rates of 10, 15 and 20 mm.day^−1^, respectively.

**Table 5:** Estimated cost of malaria treatment ($USD) at the lowland and midland dam at different water level drawdown rates.

	Reservoir water level	Current	Cost ($US) - Water level drawdown rates			% change from current		
			10 mm.day^−1^	15 mm.day^−1^	20 mm.day^−1^	10 mm.day^−1^	15 mm.day^−1^	20 mm.day^−1^
Lowland	70%	4,963.6	3,783.6	2,301.6	1,389.6	24	54	72
	75%	4,946.5	4,330.4	2,633.0	1,588.4	12	47	68
	80%	5,662.3	4,694.9	2,853.9	1,721.0	17	50	70
	85%	6,139.4	5,801.5	3,524.5	2,123.3	6	43	65
	90%	7,588.0	6,589.1	4,001.9	2,409.8	13	47	68
	95%	8,619.1	6,315.8	3,836.2	2,310.4	27	55	73
	100%	8,261.2	5,964.1	3,623.1	2,182.5	28	56	74
	Total	46,180	41,276	25,084	15,119	11	46	67
Midland	70%	2,945.1	1,544.2	657.6	373.9	48	78	87
	75%	2,945.1	1,544.2	657.6	373.9	48	78	87
	80%	2,285.4	1,200.4	513.7	293.9	46	78	87
	85%	2,285.4	1,200.4	513.7	293.9	47	78	87
	90%	1,728.4	910.1	392.1	226.4	47	77	87
	95%	1,728.4	910.1	392.1	226.4	47	77	87
	100%	1,304.4	689.1	299.6	175.0	47	77	87
	Total	15,222	7,998	3,426	1,963	47	77	87

### Economic cost of malaria at different water level drawdown rate scenario

The economic cost of malaria, estimated from lost working days, decreased with increasing rates of water level drawdown in both the lowland and midland dams (Table 6). At the lowland dam, the current total annual economic cost was estimated to be 444,000 lost working days. This was predicted to drop by 24, 54 and 72% as the water level drawdown rate increased from 10, 15 to 20 mm.day^−1^, respectively. At the midland dam, the current economic cost of malaria around the dam was estimated to be 137,951 which reduced by 47, 77 and 87% when water level drawdown rate increased from 10 to 15 and 20 mm.day^−1^, respectively.

**Table 6:** Estimated seasonal economic costs during the main malaria transmission season in terms of lost working days.

	Reservoir water level	Cost ($US) Water level drawdown rates				% change from current		
		Current	10 mm.day^−1^	15 mm.day^−1^	20 mm.day^−1^	10 mm.day^−1^	15 mm.day^−1^	20 mm.day^−1^
Lowland	70%	44,827.4	34,289.3	20,858.3	12,593.2	24%	53%	72%
	75%	51,314.2	39,244.5	23,861.5	14,395.1	24%	53%	72%
	80%	55,638.4	42,547.7	25,863.4	15,596.2	24%	54%	72%
	85%	68,766.0	52,575.7	31,941.1	19,242.8	24%	54%	72%
	90%	78,110.2	59,713.7	36,267.1	21,838.4	24%	54%	72%
	95%	74,867.4	57,236.5	34,765.8	20,937.6	24%	54%	72%
	100%	70,695.9	54,050.0	32,834.5	19,778.9	24%	54%	72%
	Total	444,219.6	339,657.4	206,391.7	124,382.1	24%	54%	72%
Midland	70%	26,690.2	13,994.6	5,959.4	3,388.2	48%	78%	87%
	75%	26,690.2	13,994.6	5,959.4	3,388.2	48%	78%	87%
	80%	20,711.6	10,878.5	4,655.0	2,663.5	47%	78%	87%
	85%	20,711.6	10,878.5	4,655.0	2,663.5	47%	78%	87%
	90%	15,663.5	8,247.4	3,553.6	2,051.6	47%	77%	87%
	95%	15,663.5	8,247.4	3,553.6	2,051.6	47%	77%	87%
	100%	11,821.0	6,244.6	2,715.2	1,585.8	47%	77%	87%
	Total	137,951.6	72,485.5	31,051.3	17,792.3	47%	77%	87%

### Model validation

**Figure 3:**
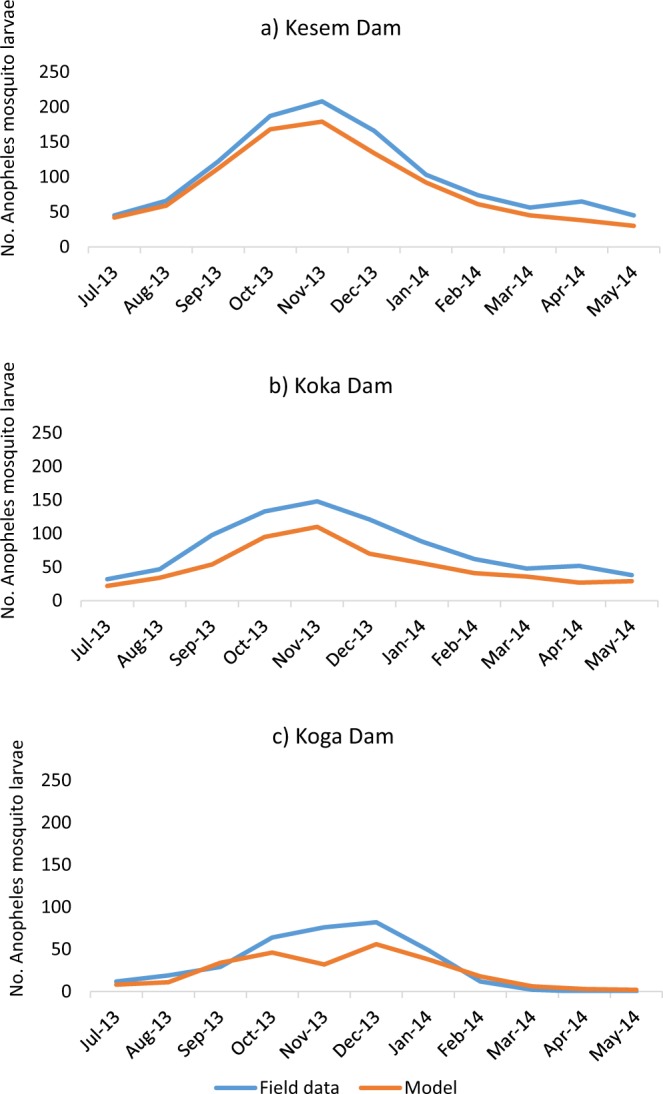
Model validation: mosquito larval abundance around Kesem, Koka and Koga dams and model predictions.

Larva abundance was compared between field data and the model (Fig. 3). The model prediction was in agreement with the actual field data. Generally, the trend of monthly larval abundance in all the three dams for the specific study period aligned with the model predictions – predicting high mosquito larval abundance in October-December, immediately after the rainy season.

## Discussion

This study is the first of its kind to model the potential for optimizing reservoir water level drawdown as a malaria control measure in SSA. We identified that increasing the rate of reservoir water level drawdown during the main malaria season (when reservoir capacity is between 70 and 100%) will decrease mosquito vector abundance and malaria prevalence around large dams in SSA. As a consequence, the EIR, malaria prevalence, cost of malaria treatment and economic cost due to malaria declined with increased rates of water level drawdown in lowland and midland regions. These findings highlight that rapid rates of water level drawdown significantly reduce mosquito larval abundance and malaria transmission.

The present study showed that the potential area of larval habitat increased with reservoir capacity at the lowland dam, but not at the midland and highland dams. These differences are explained by differences in topography of these dams: the slope of the lowland dam reservoir (2%) is shallower than that of the midland (6%) and highland (5–8%) dams^37^, suggesting that as the slope get steeper an increase in water level does not necessarily translate into increased surface area. Recent study indicated that shoreline slope is the most important malaria risk factor in dam-affected geographies, explaining 41–82% of the variation in malaria incidence around reservoirs^38^. Furthermore, lower reservoir capacities revealed small-scale topographic features (islands) that increased shoreline perimeter with decreased reservoir capacity.

Previous research reported that shoreline puddles <0.5 m depth contribute 70–90% of larval vector habitats around reservoir villages at the three study dams^30^. If reservoir water levels are managed to suppress mosquito development, a significant proportion of these breeding habitats will be minimized. The present study indicated that 10, 15 and 20 mm.day^−1^ water level drawdown rates was associated with a 24, 54 and 72% reduction in larval abundance at the lowland dam and a 48, 78 and 88% reduction at the midland dam during the main malaria transmission season, respectively. These reductions translated into a 19, 48 and 65% drop in EIR in the lowland dam, and a 40, 71, 82% decline in EIR at the midland dam, respectively. In line with our findings, Gu and Novak^34^ found that a 30% coverage of targeted larval management could reduce the EIR by 70% at low transmission areas. In Zimbabwe, Geissbuhler *et al*.^39^ showed that larval management decreased EIR from 1.06 (0.64–1.77) to 0.56 (0.43–0.77) following larval reductions. The present study highlights the potential role of dam management in controlling larval abundance and malaria transmission in Africa settings. Indeed, water level management was effectively used for malaria control in rice irrigation schemes in Kenya^40,41^.

This study did not factor in the potential economic impacts from optimized dam management for malaria control on downstream irrigated-crop production at lowland and highland dams or hydropower generation potential of the midland dam. However, a previous study at the midland dam^42^ found that the application of optimized dam management for malaria control would increase total average annual electricity generation from 87.6 GWh per year to 92.3.2 GWh per year (i.e. a 5.3% increase). The net increase in energy arose as more water was released through the turbines because losses from spill, seepage, and evaporation were reduced as a consequence of more rapid drawdown of the reservoir at the end of the wet season. Moreover, water level management was also predicted to have no impact on the capacity of the reservoir to meet downstream irrigation demands, yet would reduce downstream impacts of flooding from 28 days to 24 days per annum. The overall benefits of optimized dam operations and its associated cost should be examined in light of creating better health outcomes and its direct and indirect socio-economic advantages.

It has to be noted that every dam have a unique topography and that results from one dam location cannot be directly applied to others. In our study, there was a clear difference in the lowland, midland and highland dams. Topography plays a major role in determining the formation of potential mosquito breeding habitats around dams. Nevertheless, the techniques and methodology developed here can be applied across all dams and the lessons from this research are equally applicable. Interestingly, model validation using field data indicated that our model predicted malaria mosquito abundance with similar monthly trend and comparable numbers of mosquito larvae throughout most of the year. It has to be noted that optimized reservoir management does not completely stop mosquito breeding, instead significantly reduces the availability of mosquito breeding habitats and mosquito abundance, hence impacting malaria transmission.

With over 1.1 million new annual malaria cases estimated to have originated from constructed reservoirs in SSA^7^ and over 200 dams currently planned across the region, the need for additional malaria control measures is critical. Here, we have presented how the rate of water level drawdown can positively influence larval abundance at the reservoir scale. Future studies are required to investigate the economic cost of malaria around dams in sub-Saharan Africa compared with economic losses related to optimized dam operation for malaria control. Furthermore, research is needed to evaluate the actual benefits of optimized dam management by applying proposed water level drawdown scenarios.

This study has several limitations. One of the limitations of this study was that the resolution of the DEM (30 × 30 m) used was not able to estimate reservoir parameters for a very small change in reservoir capacity. For instance, the area of wetted shoreline at the lowland dam did not change as water level increases from 70 to 75% of full supply or from 80 to 85%. This could have been resolved using a higher resolution DEM (e.g. 5 × 5 m). Given the importance of shallow shoreline habitats for mosquito breeding, and the potential for error in scaling up to reservoir-scale estimates, future modeling should use the highest resolution DEM available to best estimate the effects of water level change on shoreline larval breeding habitats and malaria risk. The study also did not factor in unemployment rate while computing the economic cost of malaria. All the inhabitants in the study area practice farming and it is difficult to estimate employment rate since all members of households except young children participate in farming activities. Further research is needed to illustrate the added value of reservoir management above and beyond the outcome of existing vector control measures.

This study highlighted the benefits of modifying reservoir management to incorporate mosquito control, which then translate into a reduced disease and increased economic savings. Current malaria control measures around dams are mainly composed of adult vector control using bed nets and indoor residual spraying. While these measures are important for reducing mosquito-human contact, the addition of larval management will further reduce the existing burden of malaria around large dams in Africa. The findings of this study support previous studies^43–45^ that suggested integrated vector control (using LLIN, IRS and LSM including reservoir management) in order to bring down the EIR to near zero.

In conclusion, while dams offer vital economic opportunities for Africa, their management should incorporate cost-effective and complementary malaria control approaches. Increasing rates of water level drawdown during peak malaria transmission season could help reduce malaria transmission by suppressing the formation of stable larval habitat required to complete this life cycle stage. Thus, optimized dam operation, when coupled with currently exiting vector control measures, could help mitigate malaria around dams.

## Supplementary information

Supplementary Information
