# Supplementary material for: Modeling reservoir management for malaria control in Ethiopia
Source: Sci Rep. 2019 Dec 2;9:18075. doi: 10.1038/s41598-019-54536-w (PMC6889458; doi:10.1038/s41598-019-54536-w)

**Modeling reservoir management for malaria control in Ethiopia**

Solomon Kibret, Darren Ryder, G. Glenn Wilson, Lalit Kumar

**Supplementary File 1:** Perimeter of the wetted shoreline at different water volume capacities around lowland, midland and highland dams in Ethiopia.


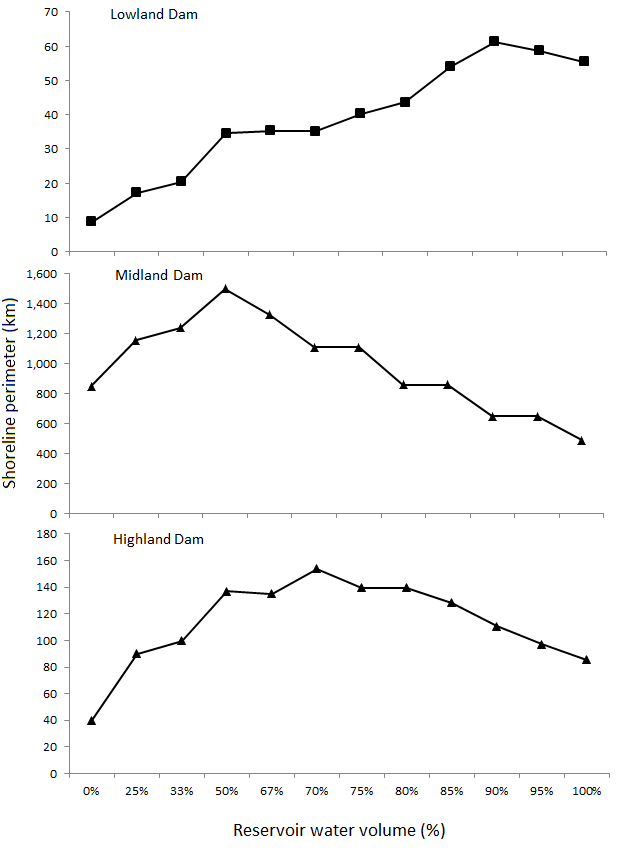

Supplement: Supplementary file 1 — Supplementary Information [file 41598_2019_54536_MOESM1_ESM.docx]
